# Chloroprocaine 3% for anesthesia during cesarean section in a patient with dopa-responsive dystonia: A case report

**DOI:** 10.1097/MD.0000000000038795

**Published:** 2024-07-05

**Authors:** Jingxin Zhou, Wenqin Zhou, Dong Luo

**Affiliations:** a Department of Anesthesiology, West China Second University Hospital, Sichuan University, Chengdu, China; Key Laboratory of Birth Defects and Related Diseases of Women and Children, Ministry of Education (Sichuan University), Chengdu, China.

**Keywords:** dopa-responsive dystonia, epidural anesthesia, levodopa/benserazide

## Abstract

**Rationale:**

Dopa-responsive dystonia (DRD) is a rare autosomal dominant hereditary disorder with a prevalence of 0.5 per million population. The disease is characterized by onset of dystonia in childhood, progressive aggravation of the dystonia with diurnal fluctuation, and complete or near complete alleviation of symptoms with low-dose oral levodopa. The incidence of DRD is low, and only a few publications have described this disorder connected with anesthesia.

**Patient concerns:**

We present a case involving a pregnant woman with DRD who continued levodopa/benserazide throughout the pregnancy. The perioperative anesthesia management was described. We used chloroprocaine 3% for epidural anesthesia during cesarean section.

**Diagnoses:**

Dopa-responsive dystonia

**Interventions:**

Levodopa/benserazide

**Outcomes:**

In summary, levodopa/benserazide was continued throughout our patient’s pregnancy with a good obstetric outcome, and chloroprocaine was safely used in epidural anesthesia without deterioration of her dystonic symptoms.

**Lessons:**

Chloroprocaine was safely used in epidural anesthesia without deterioration of her dystonic symptoms.

## 1. Introduction

Dopa-responsive dystonia (DRD), also known as Segawa disease, is a rare autosomal dominant hereditary disorder with a prevalence of 0.5 per million population. It is caused by dominant inheritance of a mutation of the guanosine triphosphate cyclohydrolase 1 gene (*GCH1*), which is located on chromosome 14q22.1-q22.2. The mutation causes a biochemical defect of tetrahydrobiopterin and may prohibit dopamine production within the cerebral basal ganglia, resulting in symptoms of DRD.^[1]^ The disease is characterized by onset of dystonia in childhood, progressive aggravation of the dystonia with diurnal fluctuation, and complete or near complete alleviation of symptoms with low-dose oral levodopa.^[2]^

We herein present a case involving a pregnant woman with DRD. The patient received levodopa/benserazide throughout her pregnancy and gave birth to a healthy female infant. We used chloroprocaine 3% for epidural anesthesia during cesarean section.

## 2. Case report

Informed consent was obtained from the patient to publish this report. A 34-year-old primipara presented for cesarean section at 38 weeks of gestation. The patient had first exhibited dystonia at the age of 4 years. The dystonia was characterized by myotonia, abnormal posture and pace, and tremor, with diurnal fluctuation, worsening in the evening and relief after sleep or rest. These symptoms progressively worsened over the years, and the condition was misdiagnosed as cerebral palsy. At the age of 20 years, a diagnostic levodopa test was positive, and the patient was diagnosed with DRD. She had taken levodopa/benserazide at 62.5 mg/d, which completely relieved her symptoms. Molecular genetic testing showed a suspected mutation of *GCH1* at the age of 28 years.

At the age of 33 years, the patient became pregnant and continued to take levodopa/benserazide at 62.5 mg/d throughout her pregnancy. If the drug was not taken in a timely manner during her pregnancy, muscle stiffness occasionally occurred. No other family members had similar symptoms. At presentation for cesarean section, the patient had normal muscle strength and tension of both upper and lower limbs, normal gait, no claudication, and slight scoliosis. She was 150 cm tall and weighed 58 kg. Airway assessment revealed Mallampati class 1 with no limitations of mouth opening or neck extension. Blood test results were normal. The fetus showed no abnormalities.

The patient’s medication was continued on the day of surgery; her regular dose of levodopa/benserazide (62.5 mg) was given 2 hours before surgery. After arrival in the operation room, one 16G intravenous line was inserted in the forearm to administer Ringer’s solution. The patient was monitored, and her blood pressure and heart rate were normal. The patient was placed in the right lateral position, and an epidural catheter was uneventfully secured in the L2/3 space. A 3-mL trial dose of lidocaine 1.5% was administered through the epidural catheter. After 5 minutes of observation, the patient showed no clinical manifestations of local anesthetic toxicity or an abnormal high block level. Next, 15 mL of chloroprocaine 3% was administered through the epidural catheter, and bilateral sensory block was achieved between T8 and S5 8 minutes later. Another 5 mL of chloroprocaine 3% was administered; 5 minutes later, the block level reached T5 to S5 and the Bromage score was 0. A healthy female infant was delivered 8 minutes after skin incision, with an Apgar score of 10 at both 1 and 5 minutes. After 40 minutes, 8 mL of chloroprocaine 3% was administered. The operation lasted for 50 minutes. The patient’s blood pressure fluctuated between 90–122/45–90 mm Hg, and her heart rate fluctuated between 70 and 85 beats/min. Bilateral transverse abdominal muscle plane nerve block was performed, and 2 mg of morphine was administered through the epidural catheter for postoperative analgesia. The total fluid infusion was 1000 mL, blood loss was 400 mL, and urine output was 200 mL. The strength and sensation of both lower limbs started recovering 20 minutes after the operation without stiffness. One hour later, the strength and sensation of both lower limbs had completely recovered and the visual analogue scale score was 1. At 24 hours after the operation, the patient could walk normally without any discomfort, and her visual analog scale score was 3. The patient and her infant were uneventfully discharged 3 days later.

## 3. Discussion

This is the first reported case in which chloroprocaine was used for a patient with DRD under epidural anesthesia and the first description of a new mutation in *GCH1* (C.557C>A,p.Thr186Lys in exon 5).

DRD is a rare disorder with a prevalence of 0.5 per million. It shows female predominance, with reported female-to-male ratios ranging from 1.3:1 to 8.3:1. The onset of dystonia in childhood and progressive aggravation with diurnal fluctuation. The classic phenotype of DRD is characterized by onset in childhood with walking difficulties due to lower limb dystonia that progresses to generalized dystonia, concurrent or subsequent development of parkinsonism (mainly rigidity and bradykinesia), which usually misdiagnosed as cerebral palsy,^[3,4]^ just like the case of our patient.

DRD is most commonly diagnosed by its characteristic clinical symptoms and complete resolution with low-dose levodopa. The phenylalanine loading test has been shown to have high sensitivity and specificity, and whole-exon gene sequencing can also be carried out. Lumbar puncture for cerebrospinal fluid examination will show decreased levels of tetrahydrobiopterin and homovanillic acid. Patients with DRD demonstrate dramatic and sustained complete resolution of symptoms to relatively low doses of levodopa regardless of the length of the clinical course. Levodopa monotherapy or levodopa/benserazide combination therapy is usually employed.^[1]^ Benserazide blocks the peripheral conversion of levodopa to dopamine, which increases bioavailability with reduced peripheral side effects. However, the treatment effect and drug selection for pregnant women with DRD remain unclear. In one reported case, administration of levodopa was discontinued because of potential teratogenic side effects.^[5]^ Watanabe and Matsubara^[6]^ indicated that levodopa should be continued during pregnancy and that levodopa monotherapy, not combination therapy, may be the better treatment choice because combination therapy increases the risk of miscarriage. Furthermore, fetal growth restriction and fetal visceral, skeletal, and intestinal malformations have been reported in pregnant women who received levodopa/benserazide.^[5]^ However, our patient continued to receive levodopa/benserazide throughout her pregnancy, and her obstetric outcome was good. Patients with DRD should consult with their neurologist and obstetrician before pregnancy to determine the treatment of choice that will most effectively balance the risks and benefits during pregnancy.

Physical examination should be conducted before anesthesia to determine whether the patient has torticollis, upper or lower limb stiffness, spasm, abnormal gait, or scoliosis. DRD may lead to pharyngeal/laryngeal dystonia, and it is necessary to observe whether the patient has difficulty speaking. Patients with increased torticollis and neck muscle tension may have limited neck mobility, which may lead to an increased risk of difficult airway. Laryngeal dystonia may lead to an increased risk of laryngeal spasm and dysphagia, which may in turn increase the risk of reflux and aspiration.^[2]^

The incidence of DRD is low, and only a few publications have described this disorder and anesthetic management. Experts have suggested that a regional anesthetic approach is preferable to general anesthesia.^[7]^ There are reports on the safe use of combined spinal/epidural anesthesia and epidural anesthesia, with bupivacaine 0.5% for spinal anesthesia and lidocaine 2% for epidural anesthesia.^[5,8]^ As the clinical guidelines of women’s and children’s health recommended, regional anesthesia is the first chose for pregnant under cesarean section. In the case, we chose epidural anesthesia with chloroprocaine for this patient, because chloroprocaine is a short-acting, amino ester-type local anesthetic which rapidly metabolized by ester hydrolysis; its duration of action is short and potential for cardiac toxicity relatively low. Chloroprocaine was safely used in this case and the sensory and motor function of the patient was completely recovered after 1 hour.^[9]^ In addition, metoclopramide which usually used before cesarean section should be avoided because the potential risk of triggering dystonia.

For patients contraindicated with intravertebral anesthesia, general anesthesia was a suitable choose for DRD patients. The safe use of propofol for intravenous general anesthesia has been reported in non-obstetric patients.^[10,11]^ In a cases series of 12 patients with DRD, the author suggested that both succinylcholine and non-depolarizing muscle relaxants were to be tolerated in these patients.^[12]^ Neostigmine and atropine have been reported to counter atracurium muscle relaxant residue in patients with DRD without significant adverse effects. Inhalation anesthetics can inhibit the synaptic reabsorption of dopamine, leading to a decreased concentration of dopamine in the brain and an increased concentration of levodopa in the blood. Halothane anesthesia in a patient being treated with levodopa will cause increased myocardial stress and lead to arrhythmia. Intravenous anesthesia may be the better choice if general anesthesia is being considered.^[5]^

In summary, in our knowledge, levodopa/benserazide was continued throughout our patient’s pregnancy with a good obstetric outcome, and chloroprocaine was safely used in epidural anesthesia without deterioration of her dystonic symptoms.

## Author contributions

**Conceptualization:** Jingxin Zhou.

**Formal analysis:** Jingxin Zhou, Dong Luo.

**Funding acquisition:** Jingxin Zhou.

**Writing – original draft:** Jingxin Zhou.

**Supervision:** Wenqin Zhou.

**Visualization:** Wenqin Zhou.

**Writing – review & editing:** Wenqin Zhou, Dong Luo.

**Software:** Wenqin Zhou.
